# The Duration of Hypotension Determines the Evolution of Bacteremia-Induced Acute Kidney Injury in the Intensive Care Unit

**DOI:** 10.1371/journal.pone.0114312

**Published:** 2014-12-12

**Authors:** Karin Janssen van Doorn, Walter Verbrugghe, Kristien Wouters, Hilde Jansens, Philippe G. Jorens

**Affiliations:** 1 Department of Nephrology-Hypertension, Antwerp University Hospital, University of Antwerp, Antwerp, Belgium; 2 Department of Critical Care Medicine, Antwerp University Hospital, University of Antwerp, Antwerp, Belgium; 3 Department of Biostatistics, Antwerp University Hospital, University of Antwerp, Antwerp, Belgium; 4 Department of Microbiology, Antwerp University Hospital, University of Antwerp, Antwerp, Belgium,; University of California Los Angeles, United States of America

## Abstract

**Background:**

Exploration of the impact of severe hypotension on the evolution of acute kidney injury in septic patients.

**Methods and Results:**

We reviewed the hemodynamic parameters of 137 adults with septic shock and proven blood stream infection in the ICU. Severe hypotension was defined as a mean arterial blood pressure (MAP) ≤65 mmHg. The influence of the duration of severe hypotension on the evolution of acute kidney injury was evaluated according to the RIFLE classification, with day 0 defined as the day of a positive blood stream infection. After bloodstream infection, the probability for a patient to be in Failure was significantly higher than before blood stream infection (OR = 1.94, p = 0.0276). Patients have a significantly higher risk of evolving to Failure if the duration of severe hypotension is longer (OR = 1.02 for each 10 minutes increase in duration of a MAP <65 mmHg, p = 0.0472). A cut-off of at least 51 minutes of severe hypotension (<65 mmHg) or at least 5.5 periods of severe hypotension within 1 day identified patients with increased risk to evolve to Failure.

**Conclusions:**

There is a significant influence of both the duration and the number of periods of severe hypotension on the evolution to Failure. Blood stream infection has a significantly negative effect on the relationship between severe hypotension and Failure.

## Introduction

Acute kidney injury (AKI) is recognized as a significant clinical problem with a high mortality and morbidity, including an increased risk of renal replacement therapy (RRT), increased progression of underlying chronic kidney disease (CKD) and prolonged hospitalization [1]. Although AKI is a syndrome comprising multiple conditions, sepsis is the major cause of AKI in the critically ill, accounting for >50% of cases [2], [3]. Outcome in AKI is influenced by the underlying disease causing the condition, the severity and duration of renal impairment and the baseline condition of the patient [3].

Acute kidney injury indeed is a complex process and it has been proposed recently to call this syndrome rather a ‘kidney attack’ [4]. The pathophysiology can be extremely complex and well beyond a single ischemic insult (toxic, allergic, metabolic, obstructive, septic). This may then lead to structural damage and/or an acute dysfunction or both. The term ‘kidney attack’ has no biochemical reference nor does it grade the severity of the insult. In practice, the diagnosis of AKI has so far been made based on changes in serum creatinine or urine output (RIFLE/AKIN). In a recent acute dialysis quality initiative consensus meeting, a new perspective has been suggested for the diagnosis of AKI (or kidney attack) including a new category of kidney disorders defined by the positivity of damage biomarkers and negativity of creatinine or urine output criteria. [4]. As the definition of AKI is still and continuously in progress, the term AKI according to the RIFLE-criteria is still used in many studies [3].

Recent consensus criteria for the definition and classification of AKI have been developed from the RIFLE criteria by the AKI Network. The AKI Network proposed several small modifications to the RIFLE criteria, with only three stages of severity, and included the additional criterion of time. Both classification systems have been validated in different populations of patients and have been shown to correlate with short-term outcomes [1], [3], [5]–[8].

A major obstacle in the management of sepsis-associated AKI is the incomplete understanding of the pathogenesis of AKI during sepsis [3], [5]. Two observations are already known: 1) in sepsis-associated AKI, the glomerular filtration rate decreases rapidly, despite preserved or increased cardiac output and hyperdynamic circulation, and 2) a delay in the administration of appropriate antimicrobials is an important independent factor associated with a higher risk of AKI [5], [6]. In contrast to the rapidly growing number of papers describing the detection of biomarkers predicting AKI, little attention has been given to the pre-analytic hemodynamic alterations that may affect progression to AKI.

The purpose of the present study was therefore to examine the impact of hypotension on the evolution of AKI in septic patients by using these validated RIFLE-criteria. Therefore, we focused on the role of hypotension as the principal objective and examined 1) the evolution of hypotension during sepsis 2) the influence of proven sepsis on the evolution to Failure and 3) the influence of hypotension on the evolution to Failure.

## Methods

### Ethics statement

The study did not interfere with the daily care or treatment of any of the patients. This observational study, without any specific intervention, was reviewed and approved by the hospital's institutional ethics board of the Antwerp University Hospital (Belgium) (reference number EC110219), and all data were anonymously processed. Informed consent was not required because this study was not interventional, but retrospective; all patients and families were, however, informed on the admission leaflet that anonymous data can be used for academic research. The data were anonymised for an optimal and objective study design. Being a retrospective study, an informed consent was not necessary. This study was not funded.

### Patient population

All adults (18 years or older) who were admitted to our intensive care unit (ICU) over a period of 36 months (1/1/2009 to 31/12/2011) were eligible for enrollment and retrospective evaluation. Only patients with proven bloodstream infection (BSI) by non-commensal flora, diagnosed according to the criteria of the Center for Disease Control [9]. (divided into three groups: gram+, gram- and yeast) and shock during their ICU stay were included. Patients with pre-existing renal failure and/or chronic dialysis were excluded from the analysis. Pre-existing renal failure was defined according to the last previous known value of “renal function” [10]. Both, creatinine and urine-output were used for fulfilling the RIFLE criteria. The last known value of serum creatinine was considered the baseline creatinine.

Most of our patients were in-hospital prior to the ICU admission and thus before they developed AKI: the last previous known creatinine value was the first documented serum creatinine value of the AKI episode and therefore used as ‘baseline’. For patients who were admitted directly with AKI to the hospital, the most recent historical creatinine value (a value known in the pre-existing medical record of our hospital or from the general practitioner) was used [10].

### Specific outcome measures

Complications and outcomes (AKI, the need for dialysis and ICU mortality) were retrieved from the electronic patient data record system (iMDsoft, MetaVision, 5.45, Tel Aviv, Israël) for each patient. Recent consensus criteria for the definition and classification of AKI have been developed from the RIFLE criteria by the AKI Network. The acronym RIFLE represents the increasing severity classes Risk (R), Injury (I) and Failure (F) and the two outcome classes Loss (L) and End-Stage Kidney Disease (E) [1]. The severity grades R, I and F are defined on the basis of changes in serum creatinine (sCr) or urinary output, wherein the worst of each criterion is used. The two outcome criteria, L and E, are defined by the duration of loss of kidney function. Given the retrospective nature of this study, AKI was assessed by the RIFLE criteria instead of the so-called AKIN classification [11].

Data were retrieved starting from 7 days before BSI was detected (BSI-7) until 7 days thereafter (BSI+7). The patients were further divided into four groups, with the predominant stage of R, I, F or no-AKI. For those patients with ICU-hospitalisation shorter than 7 days prior to BSI, the latest creatinine was taken into account as is used in all validated scores for renal failure.

In the study design, we made the decision to exclude sepsis (septic shock) without bacteremia or a microbiological source of sepsis other than caused by either gram+ or - bacteria. So on one hand, we could exclude all cases of the systemic inflammatory response syndrome as confounders. Moreover, we wanted to evaluate not only the influence of the delay of antibiotic treatment but especially if the type of bacteria (being either gram- or gram+) might attribute to the outcome. Therefore, patients solely with viable either gram- or gram+ bacteria in circulating blood were included, in order to be 100% sure on the causative bacterial agent for the infection.

In our ICU, blood is obtained from our patients at admission and at least daily (6 am). The following data were obtained: admission and laboratory values (including electrolytes, urea and creatinine), daily diuresis information and signs of infection such as white blood cells and C-reactive protein (CRP), which are routinely determined daily; demographic and clinical information, including age, gender and vital parameters; the length of stay in the ICU; and information necessary to determine the severity of illness using the Simplified Acute Physiology Score III (SAPS-3) [12] and to determine organ failure and dysfunction using the Sequential Organ Failure Assessment (SOFA) score [13].

All known and presumed causes of AKI readily and daily available in every patient in our ICU were investigated, Therefore the use of contrast agents, the daily cardiac output and all potential nephrotoxic drugs, however only other than mentioned, could not be included for all patients because of a lack of this information in some patient files for some days.

### Evaluation of hypotension

Blood pressure was in all patients measured by means of an intra-arterial (radial or femoral) line (systolic, diastolic and mean) and is stored automatically every minute in the patient data management system (PDMS). Severe hypotension was defined as a mean blood pressure ≤65 mmHg, according to the SSC guidelines [14]. All MAPs<35 mmHg were excluded. The cut-off for a MAP<35 mmHg was arbitrary chosen; it is half of the MAP used in the early goal-directed therapy targets. Besides, we decided to eliminate a MAP<35 mmHg, because this very low value may reflect an artefact, rather than a real measurement.

Moreover, none of the patients with MAP<35 mmHg but only the measurements of a MAP<35 mmHg were excluded.

Data were extracted electronically and were manually double checked by calculating the MAP according to following formula: MAP ((SBP + 2 x DBP)/3). When there was a difference of 20 mmHg between the calculated MAP ((SBP + 2 x DBP)/3) and the measured MAP by Metavision, the MAP parameter was excluded from the database.

In order to exclude artefacts, we defined a measurement one minute before and one minute after a single measurement of MAP lower than 35 mmHg as an artefact (three minutes is an artefact). After excluding these artefacts, all time periods of lower than 65 mmHg were considered for evaluation and conclusion in the analysis. Therefore a single period of hypotension can be of variable length, as long as the MAP<65 mmHg was continuously seen.

The influence of the duration of severe hypotension (defined based on the mean blood pressure in mmHg) on the evolution of AKI was evaluated according to stages R, I and F. Day 0 was defined as the day on which a positive blood culture (bacteremia) was observed. On the day of positive BSI (day 0), 3 patients presented with N-AKI, with R = 37, I = 44 and F = 53. Because of the low number of N-AKI patients, the N-AKI and R groups were considered together for further evaluation.

### Evaluation of the administration of vasopressors

The administration of diuretics and vasopressors (the validated vasopressor load (vpld) and the mean inotropic index (mii)) were also considered. We used the vasopressor load formula of Dunser: vasopressor load (µg/kg/min)  =  norepinephrine (µg/kg/min) + dopamine (µg/kg/min/kg/2) + epinephrine (µg/kg/min) + phenylephrine (µg/kg/min/10) [15].

The cumulative index of inotropic support was quantified as the total inotropic dose, as proposed by Wernovsky and colleagues [16]. The score was adapted after the introduction of milrinone [17], dopamine (µg/kg/min) + dobutamine (µg/kg/min) + 100 x epinephrine (µg/kg/min) + 100 x norepinephrine (µg/kg/min) + 20 x milrinone (µg/kg/min).

### Statistical Analysis

Numerical data were extracted from the PDMS (iMDsoft, MetaVision) by a senior ICU staff member who was blinded to each patient's status. These data were reviewed for missing information, logical errors, insufficient detail or the need for additional queries. All data were analyzed using R version 3.0.1 (R Foundation for Statistical Computing, Vienna, Austria) and SAS version 9.2 (SAS Institute, Cary, NC). Normally or nearly normally distributed variables are reported as the mean and standard deviation (SD) and were compared by Student's t test, analysis of variance or simple linear regression. Non-normally distributed continuous data are reported as medians with 25% and 75% quartiles (Q1 – Q3) and compared by the Mann-Whitney U test or Kruskal-Wallis test. Categorical data are reported as proportions and compared using the Chi-square or Fisher's exact test for unpaired comparisons and the McNemar test for paired comparisons.

The relationship between hypotension (the number of hypotensive periods and the duration of hypotension) and BSI was studied with a mixed-effects Poisson model with log-link and random-subject effects, including BSI status, RIFLE classification at admission, vasopressor load, age, gender, diabetes, SAPS3, Hb, Albumin, CRP, Lactate, SOFA score and daily fluid balance as fixed effects. By including a subject-specific intercept the correlation between measurements from the same patient is taken into account. An interaction between BSI status and RIFLE classification at admission was added to allow for different BSI effects across RIFLE classes. The coefficients and their 95% confidence intervals are reported. The effect of hypotension and/or BSI on failure was investigated by a logistic mixed-effects model with logit-link and random-subject effects. BSI status, RIFLE classification at admission, hypotension (presence and the number of periods or duration), vasopressor load, age, gender, diabetes, SAPS3, Hb, Albumin, CRP, Lactate, SOFA score and daily fluid balance were included in the model as fixed effects. The odds ratios (OR) and corresponding 95% confidence intervals are reported.

For all models, post-hoc comparisons were obtained and corrected for multiple testing using a Bonferroni correction. Additional analysis shows a high correlation between vpload and mii (+-0,8) and therefore we decided to exclude the mii from the statistical model. For the statistical models without mii, all variance inflation factors are <3. These findings minimalize the possibility of bias.

The SOFA score was excluded from the regression analysis because renal function is included in the SOFA score and we already used the RIFLE criteria in the analysis. Otherwise renal function was twice included in the statistical analysis.

A p-value<0.05 was considered significant.

## Results

### Global patient characteristics

A total of 7356 adult patients were admitted to the ICU during the study period, of whom 137 (1.9%) patients with both proven and documented sepsis and positive blood cultures (bacteremia) were eligible for further evaluation. Bacteremia occurred from 1 day before admission, up to 122 days after admission (mean 10 days, median 3 days). This number is well within the range of the reported incidence of bacteremia-proven sepsis in the ICU in Belgium [18]. Sepsis was defined using consensus criteria [14]. Patients were further divided into four groups based on their RIFLE classification on the day of positive BSI detection. Among these 137 critically ill patients, an equal distribution of age, gender and diabetes was observed for all four included groups (table 1). None of the patients had been treated with NSAIDs or ACE inhibitors prior to admission. The mean age of the population was 62 years (range 19–89 years), the mean SAPS-3 was 61.5 (range 25–94), and the mean SOFA score during the first 24 hours was 10.57 (range 3–20). The SOFA score includes both cardiovascular- and renal, but when the SOFA score was excluded from the regression analysis, there was no difference in significance from the other included parameters.

**Table 1:** Patient characteristics according to the RIFLE criteria.

		RIFLE classification on the day of a positive BSI test			
Variables	All patients (n = 137)	None/Risk (n = 40)	Injury (N = 44)	Failure (N = 53)	*p-value
Age (y)	62.23 (14.20)	59.73 (16.05)	63.57 (12.86)	63.02 (13.80)	0.6099
Gender (male)	47/137 (34.31%)	14/40 (35.00%)	16/44 (36.36%)	17/53 (32.08%)	0.9011
DM	23/137 (16.79%)	7/40 (17.50%)	9/44 (20.45%)	7/53 (13.21%)	0.6299
SAPS-3	61.52 (14.37)	55.33 (13.67)	58.86 (12.98)	68.40 (13.3)	**<0.0001**
SOFA.total	10.57 (4.22)	8.95 (3.69)	9.09 (3.78)	13.02 (3.84)	**<0.0001**
LOS.ICU (d)	15 (5–33)	16 (5–24)	13 (6–29)	21 (3–44)	0.8312
Days prior ICU (d)	4 (1–13)	4 (1–9)	3 (1–6)	7 (1–23)	**0.0491**
Mortality ICU	63/137 (45.99%)	16/40 (40.00%)	9/44 (20.45%)	38/53 (71.70%)	**<0.0001**
Diuresis.cum (ml)	1005 (250–1965)	1985 (1223–2654)	1275 (504–1930)	136 (10–564)	**<0.0001**
Diuretics	35/137 (25.55%)	15/40 (37.5%)	13/44 (29.55%)	7/53 (13.21%)	**0.0222**
Hemoglobin (g/dl)	9.68 (1.85)	9.44 (1.74)	10.12 (2.16)	9.48 (1.60)	0.2657
Albumin (g/dl)	2.44 (0.60)	2.39 (0.61)	2.47 (0.59)	2.46 (0.60)	0.8014
CRP (mg/dl)	11.9 (6.0–17.0)	10.9 (17.0–7.9)	8.1 (4.3–15.3)	12.9 (8.2–18.1)	0.1869
Lactate (mg/dl)	1.7 (1.2–3.3)	1.4 (1.1–2.0)	2.0 (1.2–3.3)	1.9 (1.4–4.1)	**0.0170**
MII	240 (0–1290)	0 (0–524)	42 (0–653)	718 (0–2868)	**0.0012**
VPload	1.00 (0.00–12.00)	0.00 (0.00–5.02)	0.00 (0.00–6.53)	10.00 (0.00–22.90)	**0.0005**
Daily Fluid Balance (ml)	680 (67–1557)	296 (−928–676)	668 (155–1684)	1044 (392–2343)	**0.0001**
Bacteria					**0.9380**
Gram +	59/137 (43.07%)	18/40 (45.00%)	17/44 (38.64%)	24/53 (45.28%)	
Gram –	61/137 (44.53%)	17/40 (42.50%)	22/44 (50.00%)	22/53 (41.51%)	
Other	17/137 (12.41%)	5/40 (12.50%)	5/44 (11.36%)	7/53 (13.21%)	

Legends for table 1:

y  =  years, d  =  days, DM  =  diabetes mellitus, SAPS-3  =  Simplified Acute Physiology Score III, SOFA  =  Sequential Organ Assessment Score, LOS  =  length of stay, Days prior ICU  =  length of hospital stay prior to ICU admission, CRP  =  C-reactive protein, MII  =  mean inotropic index, VPload  =  vasopressor load.

All laboratory values were collected on day of positive BSI. Data are presented as mean (SD between brackets) or number/total (% between brackets).

*P-value for a difference between the 3 groups (R, I and F) based on ANOVA, Kruskal Wallis test or chi-squared test.

Although the length of stay in the ICU was the same for all patient groups (table 1), ICU mortality was significantly higher in F compared with the N/R-AKI and I groups (p = 0.0043 and p<0.0001, respectively). Patients in category F also had higher SAPS-3 and SOFA scores than patients in R and I (p<0.001), also reflecting the higher pathologic severity and occurrence of organ failure in this subgroup.

Between all groups, there was no difference in the delay of antibiotic treatment, episodes of septic shock or the total number of days in septic shock (data not shown).

The groups N and R were taken together (see Table 1), because of the small population groups in both N and R and therefore table 1 gives a good overview of the stage of renal failure on the day of positive BSI. This result therefore reflects AKI at a particular moment.

In total, 75% of the study population evolved to AKI during their ICU stay, reflecting AKI during the global ICU stay and most patients evolved to F. Only 26 patients (18.7%) with AKI did not exhibit any changes in the R, I or F level during the first 5 days of their ICU stay.

If RRT was necessary, only intermittent hemodialysis was performed. During their ICU stay, 54 patients received RRT (44 of them were at that time in Failure). RRT by itself was not an evaluated parameter in this study.

In all groups, an equal number of patients was infected with either gram- or gram+ bacteria (p = 0.938, table 1). There was no significant difference between patients with gram+ or gram- infection in the occurrence of hypotension, the duration of hypotension or the number of periods of hypotension (table 2).

**Table 2:** Differences in severe hypotension on the day of positive BSI between patients with gram+ and gram- infection.

Variables	Gram + (n = 59)	Gram – (N = 61)	p-value
Hypotension (Y/N)	43/59 (72.88%)	46/61 (75.41%)	0.9142
Duration of hypotension (min)	42 (0–255)	36 (2–202)	0.9011
Number of periods (>3 min) in hypotension	4 (0–10)	3 (1–7)	0.6299

### The relationship between BSI and hypotension

On the day on which BSI was proven, hypotension occurred markedly, affecting 103 of 137 patients (75%). There was no significant influence of consecutively gram +, gram – or other pathogens on the evolution to hypotension after BSI (data not shown). The daily fluid balance is positively correlated with the duration of hypotension: the higher the fluid net positive balance, the longer the duration of hypotension and the higher the number of periods of hypotension (P<0.0001). However, when only the amount of administered fluid was taken into account, there was a significant influence on the evolution to Failure: the higher the administration of daily fluid, the lower the evolution to Failure (p<0.0001). But when the fluid administration was separately studied for crystalloid and colloid infusion, there was a significant difference in the evolution to Failure for only the use of crystalloids; the higher the amount of crystalloids, the lower the evolution to Failure (p<0.0001).

In addition, a lower Hb or albumin and a high CRP, lactate, vasopressor load or SOFA score are all significantly correlated with the duration and number of periods of hypotension (table 3).

**Table 3:** Relationship between BSI and hypotension.

	Duration of hypotension(a)		Number of periods(a)	
	Coeff (95% CI)	p-value	Coeff (95% CI)	p-value
**Type III test for fixed effects**				
BSI		0.0052		0.4430
RIFLE at admission		0.1202		0.3929
BSI x RIFLE at admission		<0.0001		0.3778
VPload (Y/N)	0.42 (0.40; 0.44)	<0.0001	0.33 (0.26; 0.40)	<0.0001
Age	0.02 (0.00; 0.04)	0.0768	0.01 (0.00; 0.03)	0.0873
Gender (M/F)	−0.27 (−0.85; 0.31)	0.3610	−0.38 (−0.79; 0.03)	0.0715
DM	0.18 (−0.57; 0.93)	0.6347	0.22 (−0.31; 0.74)	0.4178
SAPS3	0.02 (0.00; 0.04)	0.1088	0.01 (0.00; 0.03)	0.1630
Hb	−0.06 (−0.06; −0.05)	<0.0001	−0.05 (−0.07; −0.02)	0.0002
Albumin	−0.24 (−0.26; −0.23)	<0.0001	−0.27 (−0.34; −0.21)	<0.0001
CRP	0.07 (0.06; 0.08)	<0.0001	0.10 (0.06; 0.13)	<0.0001
Lactate	0.02 (0.02; 0.02)	<0.0001	−0.02 (−0.04; −0.01)	0.0002
SOFA	0.06 (0.06; 0.07)	<0.0001	0.05 (0.03; 0.06)	<0.0001
Daily Fluid Balance (per 100)	0.001 (0.001; 0.001)	<0.0001	0.006 (0.005; 0.008)	<0.0001
**Post-hoc comparisons**				
Effect of BSI, RIFLE 0 = F	−0.21 (−0.23; −0.19)	<0.0001	0.00 (−0.08; 0.08)	0.9366
Effect of BSI, RIFLE 0 = I	0.05 (0.03; 0.08)	<0.0001	−0.01 (−0.10; 0.09)	0.8648
Effect of BSI, RIFLE 0 = R/N	0.10 (0.07; 0.12)	<0.0001	0.07 (−0.02; 0.17)	0.1283

(a) Based on a multiple Poisson mixed-effects model.

### The influence of sepsis on the evolution of the RIFLE category

Patients already in F were immediately included in the population, because they have also the opportunity to evolve to a better AKI-stage (Injury, Risk or No-AKI).

After BSI, the probability for a patient to be in Failure is significantly higher than before BSI (OR = 1.94, p = 0.0276, table 4). This probability is also significantly higher for patients who were in Failure on the day of admission compared to patients in R/N (OR = 33.03, p<0.0001). Patients who were in Injury on the day of admission have a slightly lower chance of evolving to Failure, compared to patients in R/N (OR = 0.25, p = 0.0254).

**Table 4:** Influence of sepsis and hypotension on the evolution of the RIFLE criteria.

	P (Failure)(a)		P (Failure)(a)	
	Hypotension: duration (per 10 min)	Hypotension: number of periods	
	OR (95% CI)	p-value	OR (95% CI)	p-value
BSI (Y/N)	1.94 (1.08–3.50]	0.0276	2.01 [1.11; 3.63]	0.0204
Hypotension	1.02 (1.00–1.03]	0.0472	1.02 [0.99; 1.05]	0.2009
RIFLE at admission		<0.0001		<0.0001
RIFLE 0 = F(b)	33.03 (8.89–122.77)	<0.0001	35.15 (9.31; 132.73)	<0.0001
RIFLE 0 = I(b)	0.25 (0.08–0.84)	0.0254	0.25 (0.08; 0.86)	0.0273
VPload (Y/N)	0.43 (0.20–0.93)	0.0310	0.44 (0.21; 0.96)	0.0402
Age	1.01 (0.97–1.05)	0.5555	1.01 (0.97; 1.05)	0.5916
Gender (M/F)	0.85 (0.28–2.55)	0.7670	0.83 (0.27; 2.52)	0.7360
DM	0.71 (0.17–2.91)	0.6342	0.73 (0.17–3.04)	0.6608
SAPS3	1.01 (0.97; 1.05)	0.5068	1.01 (0.97; 1.05)	0.5127
Hb	1.09 (0.87–1.35)	0.4627	1.08 (0.87; 1.35)	0.4744
Albumin	0.84 (0.44–1.61)	0.5941	0.81 (0.42; 1.56)	0.5210
CRP (per 10)	0.86 (0.58–1.26)	0.4380	0.87 (0.59; 1.28)	0.4748
Lactate	1.06 (0.88–1.27)	0.5468	1.09 (0.91; 1.31)	0.3690
SOFA	1.35 (1.21–1.52)	<0.0001	1.36 (1.21; 1.52)	<0.0001
Daily Fluid Balance (per 100)	1.01 (0.99–1.03)	0.1789	1.01 (0.99; 1.03)	0.2102

(a) Based on a logistic mixed-effects model. The interactions between BSI, duration of hypotension and RIFLE 0 were not significant.

(b) Compared with RIFLE 0  =  R/N.

### How does severe hypotension influence evolution to Failure?

**Figure 1:**
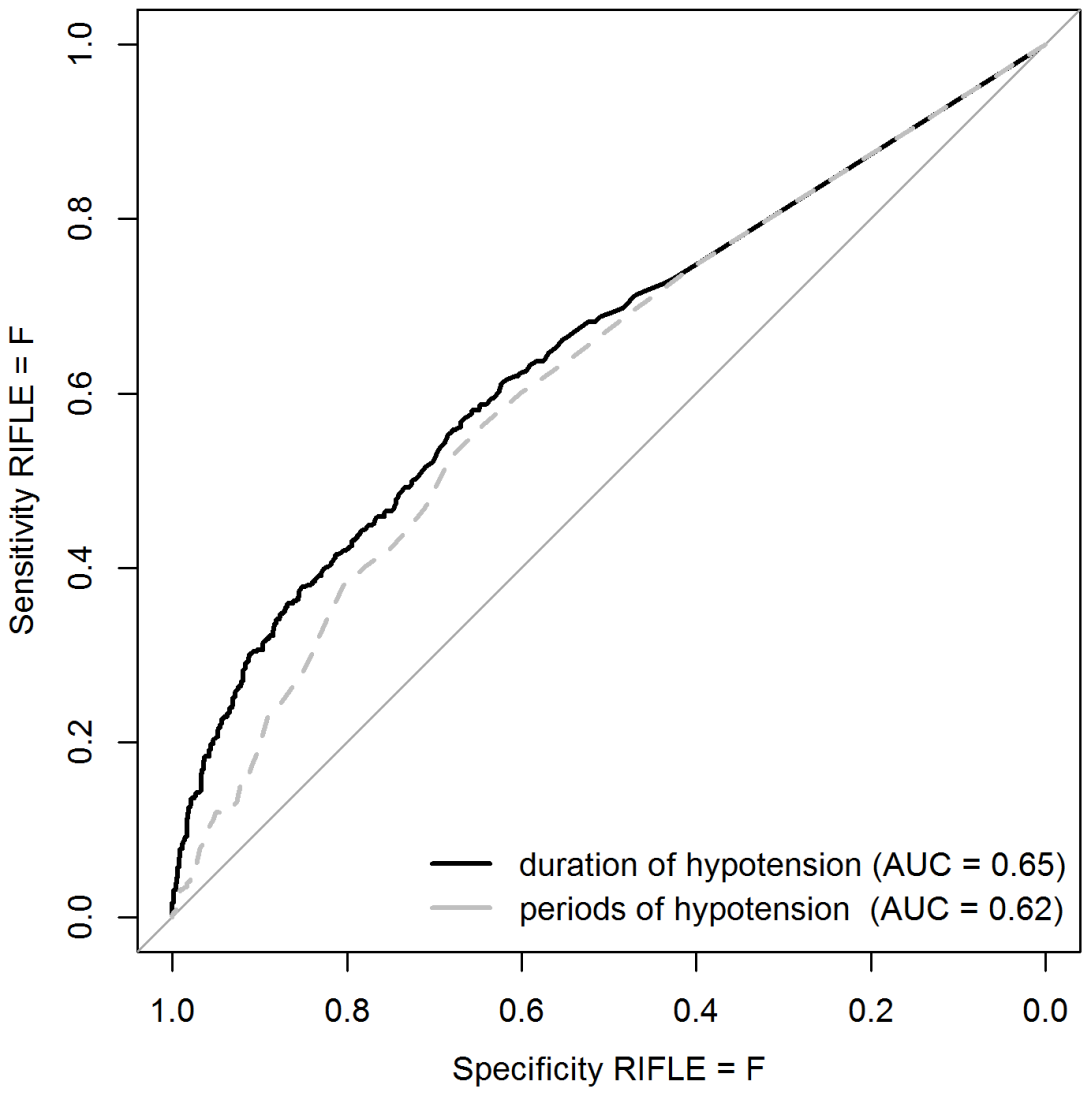
ROC curve for the detection of RIFLE  =  F based on the duration or number of periods of severe hypotension.

Patients have a significantly higher risk of evolving to F if the duration of severe hypotension is longer (OR = 1.02 for 10 minutes increase in duration of hypotension is, p = 0.0472). Fig. 1 shows the ROC curve for the detection of RIFLE  =  F based on the duration or the number of periods of severe hypotension. In our population of septic patients, a cut off of at least 51 minutes of severe hypotension (<65 mmHg (Sens = 0.55, spec = 0.68)) or at least 5.5 periods of severe hypotension within 1 day (Sens = 0.52, spec = 0.68) was determined to identify patients with increased risk of evolving to Failure.

## Discussion

Sepsis represents a continuum beginning with a host-pathogen interaction that triggers a complex interplay between pro-inflammatory, anti-inflammatory and apoptotic mediators. As the disease progresses, organ dysfunction can result from circulatory insufficiency due to hypovolemia, myocardial depression, increased metabolic demands and vasoregulatory perfusion abnormalities. These hemodynamic perturbations lead to an imbalance between systemic oxygen supply and demand, leading to global tissue hypoxia and shock [19].

AKI is a common clinical problem in ICU patients, and it independently predicts poor outcome [1], [3], [20]–[22]. The etiology of AKI in critically ill patients is often multi-factorial. However, sepsis has been consistently found to be a leading contributing factor in AKI during critical illness [1], [20]–[21]. The discrimination between septic and non-septic AKI may have clinical relevance. Septic AKI is characterized by distinct pathophysiology and has different clinical outcomes and responses to interventions compared with non-septic AKI. AKI also has a negative impact on the long-term mortality of hospital-surviving septic patients [1], [23]–[24]. Recently, the progression of the RIFLE classification and 28-day mortality in patients with severe sepsis and septic shock was analyzed. Newly developed AKI or progression from R to a higher RIFLE class during an ICU stay, and not the RIFLE class at admission, was independently associated with an increased risk of mortality [25]. Our results, which showed that septic patients with proven bacteremia in class R/N have a significantly higher risk of evolving to F, add to these previous results.

In patients with sepsis, pre-renal factors significantly contribute to AKI. Despite optimal hemodynamic monitoring, rapid hemodynamic resuscitation and intravascular volume restoration according to the Surviving Sepsis Campaign Guidelines [14], certain patients remain hypotensive. In such patients, the autoregulation of renal blood flow can be lost, contributing to AKI. A higher mean arterial pressure due to vasopressor and/or inotropic drugs might raise the glomerular filtration rate.

The duration of hypotension before initiation of effective microbial therapy is a critical determinant of survival in human septic shock [26]. Despite this clear observation, the literature on the exact hemodynamic goals to be applied in a clinical setting is scarce. There are no randomized controlled studies on the effects of different blood pressure levels on outcome. However, limited data from small cohort studies suggest a sort of consensus on the use of arterial blood pressure targets in sepsis, and the preferred target range is 65–75 mmHg [27]. In this study we preferred a MAP target of 65 mmHg because the most recent SSC guidelines recommend that vasopressor therapy initially target a MAP of 65 mmHg (grade 1C) [14]. This was underlined by an observational study which demonstrated an association between good clinical outcome in septic shock and MAP≥65 mmHg [28]. Recently, Dünser et al investigated the association between the arterial blood pressure and mortality in sepsis and concluded that a MAP level ≥60 mmHg may be as safe as higher MAP levels during the first 24 h in septic patients. The group also hypothesized that although a MAP of 60 mmHg does not influence mortality, a higher MAP may be required to maintain kidney function [29]. This hypothesis is confirmed by our study, which showed a significant influence of severe hypotension (<65 mmHg) on the evolution to Failure. What might be the underlying mechanism for this observation? In patients admitted with community acquired pneumonia, arterial hypotension over the first three days is associated with markers of monocyte deactivation. The duration of exposure to hypotension may be more important than the magnitude, and monocyte deactivation correlates with interleukin-6 and interleukin-10 release. These results suggest that persistent hypotension might contribute to immunosuppression following septic shock [30]. We also observed that the duration of hypotension seemed more important than the number of hypotensive periods in critically ill patients with proven BSI.

Recently, Poukkanen et al. reported on the hemodynamic variables and progression of AKI in critically ill patients with severe sepsis; the so called FINNAKI study [31]. The authors investigated in this prospective, observational study the progression of AKI within the first 5 days of ICU admission defined as new onset or worsening of AKI, according to the KDIGO guidelines, for both the AKIN/RIFLE classification. Although, this study has a different design than ours, - they converted all MAP data into 10-minute median (MAP) values and we used every-minute MAP - they also came to the conclusion that patients with progression of AKI had significantly lower time-adjusted MAP, 74.4 mmHg [68.3–80.8], than those without progression, 78.6 mmHg [72.9–85.4], p<0.001. A cut-off value of 73 mmHg for time-adjusted MAP best predicted the progression of AKI. Moreover, only the duration of a MAP<60 mmHg had a highly significant bad influence on the progression of AKI [31]. These data are in agreement with our findings of the influence of the duration of hypotension on the progression of AKI.

In humans with septic shock, hypotension is treated by the use of vasopressors and/of the intravenous administration of fluids. Vasopressor therapy remains a cornerstone of the clinical treatment of septic shock [32]. A beneficial renal effect of norepinephrine in septic patients was reported by Albanèse and colleagues [33]. In another study, Gordon et al used the RIFLE criteria to compare vasopressin with norepinephrine in patients with septic shock. Vasopressin was associated with a lower rate of progression to the Failure or Loss categories and a lower rate of use of RRT compared with norepinephrine. Half of the patients in R had deterioration in renal function. Interestingly, it was in the Risk subgroup of patients that a beneficial effect of vasopressin treatment was observed [34].

Beyond vasopressors, the administration of intravenous fluids during initial resuscitation for severe sepsis and septic shock to achieve a mean pressure higher than 65 mmHg is recommended [14], [35]. On the other hand, aiming for higher MAP levels during the initial resuscitation of patients in septic shock increases exposure to fluids, vasopressors and inotropics, and such exposure has been associated with increased mortality [15], [36]. Although MAP is a cornerstone of the hemodynamic management of patients with septic shock, the optimal target value remains unknown. Recently, in an experimental model that fulfills the criteria for a clinically relevant model, Corrêa et al tested whether different titrations of mean arterial pressure (50–60 and 75–85 mmHg) would have different effects on sepsis-related organ dysfunction [36]. In this study, a higher blood pressure window was associated with an increased need for fluid resuscitation and norepinephrine support. However, titrating the lower blood pressure range coincided with an increased incidence of AKI. In contrast, neither the inflammatory response nor tissue mitochondrial activity showed any difference. This study demonstrated that any standard resuscitation strategy may be a double-edged sword with respect to various therapeutic endpoints [36], [37].

Our clinical study underscores this observation that low MAP levels are associated with a higher incidence of AKI [36], with the added finding that the vasopressor load and inotropic index were the same for all patients. This was confirmed by other observational studies [38], and a higher fluid balance and lower urine volume appear to be independent predictors of mortality in AKI [39]. In resuscitated, critically ill patients, the distribution volume of serum creatinine (sCr) is higher, which may lead to underestimation of the severity of AKI and increase the time required to identify an increase in sCr. This information is used for staging AKI according to the RIFLE criteria, particularly in settings in which sCr is increasing relatively slowly, as might be expected in elderly patients with a low body mass index (BMI) [40]. Fluid overload can be managed by diuretics, but the role of diuretics during septic shock is still controversial [38]. In our study population, the use of diuretics was higher in the R-group. Moreover according to the SSC guidelines, resuscitation with crystalloids is recommended [14]. Our study underlines this advocacy, because using crystalloids protected for a worse evolution of AKI, at least in our study population.

Our study had certain limitations. We assessed vasopressor load and the mean inotropic index and did not examine the specific renal effects of these different drugs separately. In addition, cardiac output and several potential toxic drugs could not be included for all patients because of a lack of this information in some patient files and were therefore not included in the results. In contrast, the present retrospective, observational study had sufficient strength such that we could use both urine output and the sCr to fulfill the RIFLE classification. Moreover, we only included patients with proven bacteremia. In addition, we determined important answers to the study questions as shown in the results section and conclusions.

## Conclusions

*First*, in our population, patients who were in Injury on the day of admission do not have a significant risk of evolving to Failure, compared to patients in R/No-AKI.

*Second*, there is a significant influence of both the duration and the number of periods of severe hypotension (<65 mmHg) on the evolution to Failure.

*Third*, a positive fluid balance has a significant positive effect on the evolution to Failure.

*Fourth*, additional and prospective studies should be performed to develop criteria for determining an individually adapted arterial pressure threshold to preserve renal function in patients in septic shock.
